# High throughput proteomics identifies a high-accuracy 11 plasma protein biomarker signature for ovarian cancer

**DOI:** 10.1038/s42003-019-0464-9

**Published:** 2019-06-20

**Authors:** Stefan Enroth, Malin Berggrund, Maria Lycke, John Broberg, Martin Lundberg, Erika Assarsson, Matts Olovsson, Karin Stålberg, Karin Sundfeldt, Ulf Gyllensten

**Affiliations:** 1 Department of Immunology, Genetics, and Pathology, Biomedical Center, Science for Life Laboratory (SciLifeLab) Uppsala, Box 815, Uppsala University, SE-75108 Uppsala, Sweden; 2 Department of Obstetrics and Gynaecology, Institute of Clinical Sciences, Sahlgrenska Academy at Gothenburg University, Gothenburg, Sweden; 3 OLINK Proteomics, Uppsala Science Park, SE-751 83 Uppsala, Sweden; 4 Department of Women’s and Children’s Health, Uppsala University, Uppsala, Sweden

**Keywords:** Ovarian cancer, Diagnostic markers, Machine learning

## Abstract

Ovarian cancer is usually detected at a late stage and the overall 5-year survival is only 30–40%. Additional means for early detection and improved diagnosis are acutely needed. To search for novel biomarkers, we compared circulating plasma levels of 593 proteins in three cohorts of patients with ovarian cancer and benign tumors, using the proximity extension assay (PEA). A combinatorial strategy was developed for identification of different multivariate biomarker signatures. A final model consisting of 11 biomarkers plus age was developed into a multiplex PEA test reporting in absolute concentrations. The final model was evaluated in a fourth independent cohort and has an AUC = 0.94, PPV = 0.92, sensitivity = 0.85 and specificity = 0.93 for detection of ovarian cancer stages I–IV. The novel plasma protein signature could be used to improve the diagnosis of women with adnexal ovarian mass or in screening to identify women that should be referred to specialized examination.

## Abstract (web summary)

Stefan Enroth et al. identify an 11 plasma protein signature that accurately distinguishes between ovarian cancer and benign tumors. The signature was identified after measuring 593 plasma proteins in three cohorts and could be used in the future to improve diagnosis or identify women who should be referred to a specialist.

## Introduction

Ovarian cancer is currently the 7th most common cancer across the world with estimated incidences from 4.1 to 11.4 cases per 100,000 women^1^. Since ovarian cancer is commonly caught late, the overall 5-year survival rate is only 30–40%. MUCIN-16 (also known as Cancer antigen 125, CA-125) was introduced as a biomarker for ovarian cancer in 1983^2^ and is currently the most important single biomarker for epithelial ovarian cancer managment^3^. MUCIN-16 alone, however, has low sensitivity for early-stage cancer (50–62%) at a specificity of 94–98.5%^3^. The difficulties in establishing highly accurate early diagnoses with non-invasive methods, combined with the low survival rate, justifies that women with a transvaginal ultrasound (TVU) indication of adnexal ovarian mass are commonly diagnosed by surgical sampling. However, the degree of surgical over-diagnosis is high. Among women that were diagnosed by surgical sampling, only 21–30% have OC stage I–IV, while 58% have been reported to have benign tumors and the remaining 15% borderline tumors^4–6^. According to the Swedish GynOpRegistry statistics for 2017, 13% of the women with adnexal ovarian mass that underwent surgery developed complications related to the procedure^7^. A non-invasive diagnostic test with higher sensitivity and retained specificity that distinguishes between women with malignant and benign ovarian adnexal mass could be used to avoid over-diagnostic surgery. Application of MUCIN-16 and other biomarkers, including WFDC2 (WAP Four-Disulfide Core Domain 2, also known as HE4—human epididymal protein 4), such as in the ROMA Score (Ovarian Malignancy Risk Algorithm), can increase the sensitivity to 94.8% at a specificity of 75%^8^ in patient cohorts with predominantly (74.6%) late stage (III and IV) ovarian cancers. However, the low sensitivity for detection of early-stage ovarian cancer still prohibits population screening using current biomarker tests. A recent study in the UK suggests that multi-modal tests are approaching sufficient accuracy to justify screening from a health-economic stand-point^9^. However, tests with low specificity have a high false positive rate, which will result in unnecessary anxiety and examinations and also an additional cost for the health-care system.

The presently available biomarkers are mainly used to improve diagnosis of women that experience symptoms or when imaging such as TVU or computer tomography (CT) indicate adnexal ovarian mass. The tests/algorithms then triage patients in need of surgery at tertiary cancer centers. Even in this context, identification of clinically useful biomarkers based on single or combination of proteins is challenging. Recent developments of high-throughput technologies for detection and quantification of proteins has made it possible to study thousands of biomarker candidates in a single sample. Skates and colleagues^10^ have presented a statistical framework for study design, sample size calculation in discovery and replication stages and for identification of single biomarkers that can distinguish between cases and controls, with special reference to ovarian cancer. They recommend selection of the highest ranking 50 biomarkers from a discovery stage, which are then examined in a replication stage. A smaller set of replicated markers is then used to build a classifier that is tested in clinical validation studies. We have previously shown^11^ that plasma protein levels for several protein biomarkers are highly correlated. This implies that sets of proteins can be identified in a discovery stage whose combined predictive power is not greater than their individual contribution. Also, biomarkers that are not significant on their own can increase the predictive power in combination with other, individually significant or non-significant, biomarkers. An alternative approach to the framework presented by Skates^10^ is to use multivariate methods from the start, searching for combinations of biomarkers that separate cases from controls. Sample size estimates based on statistical power in relation to prediction models with linear regression is however not straightforward, and several suggestions have been presented^12–15^. All these methods rely on a range of assumptions on underlying distributions of the variables and outcome, the number of variables and expected correlation between the predicted outcomes and the actual outcomes. These factors are commonly unknown *a priori*, making such calculations difficult before the discovery stage.

One approach for finding optimal combinations of highly predictive biomarkers is to use exhaustive searches, such as the approach taken by Han and colleagues^16^, where 165 combinations of MUCIN-16 and a selection of three out of 11 additional biomarkers were examined for their ability to separate high-grade serous ovarian carcinoma from benign conditions. Such exhaustive approaches quickly become computationally unfeasible when the number of candidate proteins is high. For instance, choosing 4 from 1000 proteins can be done in over 40 billion ways. Another strategy is to use feature selection with machine learning frameworks to select subsets of informative markers from a larger set. Such approaches have previously been used to construct a classifier with 9 proteins selected from 299 in cyst-fluid separating Type 1 and Type 2 ovarian cancers^17^, or to build a classifier with 12 biomarkers selected from 92 in sera, separating ovarian cancer from healthy controls or benign conditions^18^. This is achieved by splitting the samples into a training and a test set, but with fairly small sample sizes different models are usually generated depending on the subset of samples used for training. To overcome these limitations, we developed a novel analysis strategy based on building models separating ovarian cancer from benign tumors, where we first identify smaller sets of proteins that are robustly selected across several splits, so-called cores. In the second step, we build a model by extending a core with additional proteins that have high predictive power in combination with the specific core.

Here, we aim to identify multiple mutually exclusive biomarker signatures differentiating benign conditions from ovarian cancers at different stages, grades and all histological subtypes. The signatures should be practically useful and therefore contain up to 20 proteins selected from a total of 593 characterized plasma proteins in one discovery cohort and two replication cohorts. We finally identify one model based on 11 biomarkers and age that we implement as a custom multiplex PEA assay reporting in absolute concentrations, and validate its performance in a third independent cohort.

## Results

### Characterization of plasma proteins

A total of 552 proteins were characterized in the discovery cohort (*n* = 169, Table 1) and two replication cohorts (*n* = 248, Table 1) using the proximity extension assays (PEA) with 6 of the Olink Proseek panels (Cardiometabolic, Cell Regulation, Development, Immune Response, Metabolism, and Organ Damage) (Methods). These measurements were combined with a previous study^19^ containing data from 5 PEA panels, 460 proteins, in the discovery cohort, bringing the total number of unique proteins included in the analysis to 981. Forty-two of the 460 proteins have also been quantified in the replication cohorts using the proximity extension assay in two custom 21-plex panels as previously described^19^. Following quality controls and normalization (Methods), a common set of 593 proteins (42 proteins from the previous 5 panels and 551 from the additional 6 panels) characterized in all samples were used.

**Table 1:** Cohort statistics

Cohort	Origin^a^	Types	No of Women	Age, mean (SD)	CA-125^b^
Discovery	Gbg	Benign tumors	90	60.0 (16.8)	16.8 (9.9)
		Stage I–II	42	60.7 (12.4)	67.6 (72.0)
		Stage III–IV	37	63.8 (14.1)	327.4 (284.5)
1^st^ Replication	Gbg	Benign tumors	71	60.2 (14.5)	NA
		Stage I–II	44	62.4 (13.7)	NA
		Stage III–IV	56	61.6 (11.3)	NA
2^nd^ Replication	UCAN	Stage I–II	13	55.9 (15.0)	NA
		Stage III–IV	64	59.4 (12.0)	NA
3^rd^ Replication	Gbg	Benign tumors	106	57.9 (16.1)	31.5 (29.7)
		Borderline	28	49.4 (19.6)	58.0 (50.4)
		Stage I–II	25	65.2 (10.0)	96.5 (116.4)
		Stage III–IV	65	61.4 (12.2)	739.0 (812.5)

^a^UCAN: collection at Uppsala Biobank, Uppsala University, Sweden. Gbg: Gynaecology tumor biobank at Sahlgrenska University Hospital, Göteborg, Sweden

^b^Measured at clinic [U/L], median (median absolute deviation). NA indicates ‘not available’

### 484 distinct predictive models for ovarian cancer

**Fig. 1:**
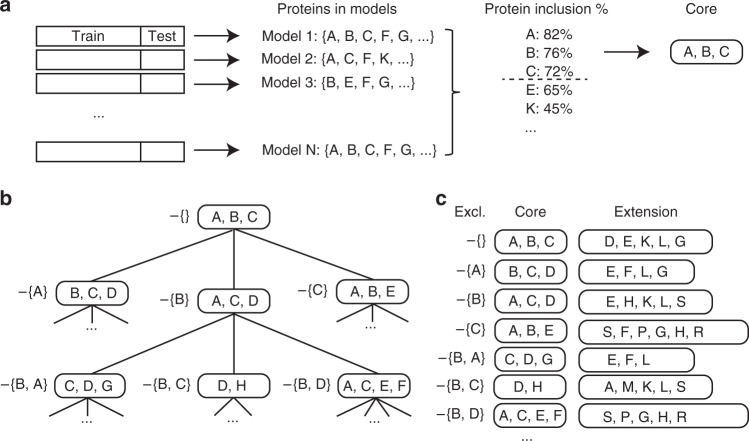
Model Generation. (**a**) Repeated model generation over random splits of the data. Proteins present in a sufficient fraction of the models are included into the core. **b** Generation of mutually exclusive cores. Proteins present in the first core (top node) are sequentially withheld from the second round of core discovery, as indicated by the sets to the left of the nodes. Each core of size N generates N new search-branches. **c** The final models are built by adding proteins to each core. The added proteins are chosen with respect to the proteins excluded in the core-discovery. Proteins are added in a stepwise forward selection choosing the protein that explains the highest proportion of remaining variance in the decision. See Methods for details

**Fig. 2:**
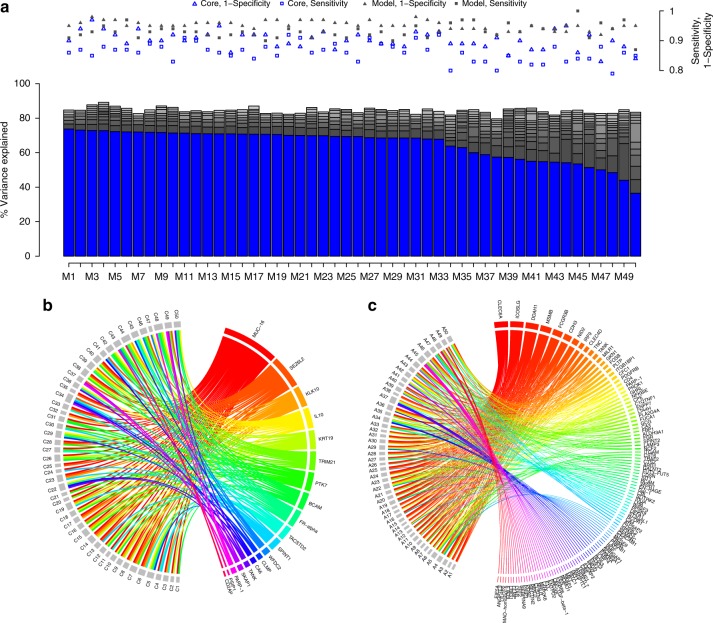
Top 50 model characteristics. **a** Variance explained in the decision (Benign tumor or ovarian cancer stage III–IV by the cores (as indicated in blue) and by the additional proteins (gray) in the test set of the Discovery Data. Sensitivity and 1-Specifity of the cores (hollow markers) and the full models (filled markers) are shown (right axis) in red. (**b**) Protein inclusion into cores. Top 50 cores are indicated with C1, …, C50 and proteins are labeled with their short name. A connector represents inclusion of that protein in a core. **c** Same as (**b**) but for additional proteins (not including core-proteins). Top 50 additional protein-sets are indicated by A1, …, A50

Models were generated using only the discovery data, according to our two-stage strategy. First, mutually exclusive protein cores, consisting of a smaller set of proteins, were selected by repeatedly splitting the data into training and test sets and retaining proteins that were present in at least 70% of the models (Methods, Fig. 1a, b). Additional biomarkers were subsequently added to each core using a stepwise forward selection approach (Methods, Fig. 1c). The addition of proteins was terminated when the total model size was 20 proteins, or the next protein to be added did not substantially increase the performance of the model (Methods). Using this strategy, we generated models to distinguish benign tumors from ovarian cancer stages I–II, III–IV, and I–IV with focus on either sensitivity, specificity or both (Methods). This analysis resulted in 484 unique models with at least one protein not overlapping between each pair of models (mutually exclusive protein signatures). The individual performance in the test-partition of the discovery data for the highest ranking 50 models is shown in Fig. 2a. MUCIN-16, which is the clinically most useful single biomarker today, was the most common protein across cores in the 50 highest ranking models by sorting on their average sensitivity and specificity in the test set from the discovery data (Fig. 2b). Our search strategy specifically excludes sets of protein, and 448 of the detected cores did not contain MUCIN-16. In general, when MUCIN-16 was not included, the models contained a higher number of proteins (9–20) than when it was included (8–17). In total, 371 proteins were included in a core, or as an additional protein in at least one model. Among the top-ranking 50 cores and models, 19 proteins made up the core-set and additional 115 proteins were selected in the addition phase (Fig. 2b, c). The performance of the 484 models in the test data is listed in Table 2 and a complete account of the models and their performances are listed in Supplementary Data 2.

**Table 2:** Performance ranges of all models

Stage^a^	MUC16^b^	No.	Size	Cohort	AUC	PPV	NPV	BPsens^c^	BPspec^c^	FSEse^d^	FSEsp^d^	FSPse^d^	FSPsp^d^
I–II	Yes	36	8–17	Discovery	0.80–0.94	0.71–0.89	0.89–0.97	0.77–0.95	0.84–0.93	0.99–1.00	0.04–0.14	0.58–0.90	0.95–0.96
				1st Repl.	0.58–0.71	0.55–0.69	0.75–0.81	0.63–0.74	0.68–0.80	NA	NA	0.16–0.45	0.94–0.95
				2st Repl.	0.49–0.83	0.30–0.59	0.92–0.98	0.74–0.92	0.68–0.83	1.00–1.00	0.06–0.06	0.12–0.51	0.94–0.95
	No	448	9–20	Discovery	0.54–0.91	0.44–0.84	0.76–0.94	0.60–0.89	0.61–0.91	1.00–1.00	0.04–0.07	0.13–0.77	0.95–0.96
				1st Repl.	0.46–0.82	0.50–0.77	0.69–0.89	0.53–0.83	0.64–0.84	0.99–1.00	0.06–0.09	0.16–0.59	0.94–0.96
				2st Repl.	0.41–0.93	0.27–0.78	0.89–1.00	0.71–0.98	0.63–0.92	1.00–1.00	0.05–0.06	0.08–0.81	0.94–0.95
III–IV	Yes	36	8–17	Discovery	0.95–0.96	0.94–1.00	0.98–1.00	0.95–1.00	0.97–1.00	1.00–1.00	0.04–0.10	0.93–1.00	0.95–0.96
				1st Repl.	0.85–0.92	0.82–0.93	0.88–0.93	0.84–0.91	0.86–0.95	0.97–0.98	0.11–0.31	0.68–0.86	0.95–0.96
				2st Repl.	0.75–0.91	0.76–0.92	0.77–0.92	0.74–0.90	0.79–0.93	0.95–0.96	0.15–0.50	0.50–0.82	0.94–0.96
	No	448	9–20	Discovery	0.94–0.96	0.89–1.00	0.97–1.00	0.93–1.00	0.95–1.00	0.99–1.00	0.04–0.12	0.90–1.00	0.95–0.96
				1st Repl.	0.78–0.90	0.78–0.95	0.82–0.92	0.76–0.91	0.80–0.96	0.96–0.99	0.07–0.34	0.54–0.87	0.94–0.96
				2st Repl.	0.77–0.94	0.77–0.96	0.77–0.97	0.74–0.97	0.78–0.97	0.95–0.97	0.19–0.69	0.42–0.92	0.94–0.96
I–IV	Yes	36	8–17	Discovery	0.88–0.94	0.88–0.95	0.86–0.96	0.85–0.95	0.89–0.96	0.95–0.96	0.32–0.74	0.76–0.93	0.95–0.96
				1st Repl.	0.75–0.83	0.83–0.89	0.69–0.75	0.73–0.80	0.77–0.87	0.95–0.96	0.09–0.24	0.47–0.65	0.95–0.96
				2st Repl.	0.70–0.87	0.75–0.89	0.70–0.87	0.71–0.87	0.73–0.89	0.95–0.95	0.14–0.59	0.39–0.73	0.95–0.96
	No	448	9–20	Discovery	0.74–0.92	0.76–0.93	0.76-0.90	0.70–0.88	0.79–0.93	0.95–0.96	0.04–0.55	0.49–0.84	0.95–0.96
				1st Repl.	0.67–0.84	0.78–0.92	0.60–0.80	0.62–0.83	0.73–0.90	0.95–0.96	0.04–0.35	0.35–0.72	0.95–0.96
				2st Repl.	0.75–0.93	0.77–0.95	0.73–0.96	0.74–0.96	0.75–0.95	0.95–0.96	0.16–0.83	0.41–0.91	0.94–0.96

All ranges indicate lowest and highest values for all models on that row

‘NA’ means that not such point exists

^a^Performances are for benign tumors vs this stage of ovarian cancers

^b^Indicates whether or not Mucin-16 was included in the model

^c^Performances when cut-off is chosen at the best point (BP, closest point on ROC-curve to perfect classification)

^d^Performances at a point on the ROC-curves with at least 0.93 sensitivity (FSEse and FSEsp) or specificity (FSPse and FSPsp)

### Model performances in the replication cohorts

The performance of each model created from the discovery data was then evaluated in two replication cohorts (see Methods). The performance ranges of the models are shown in Table 2. The top-ranking models all contained MUCIN-16, but overall, the average performance of models with MUCIN-16 did not display any pattern in terms of improved result relative to those without MUCIN-16. About one third of the performance measurements showed statistically higher scores in models with MUCIN-16, about one-third had lower scores and the last third did not show any significant difference in score (Wilcoxon ranked sum test, Bonferroni adjusted *p*-values, Supplementary Data 3).

### Top-ranking model

**Fig. 3:**
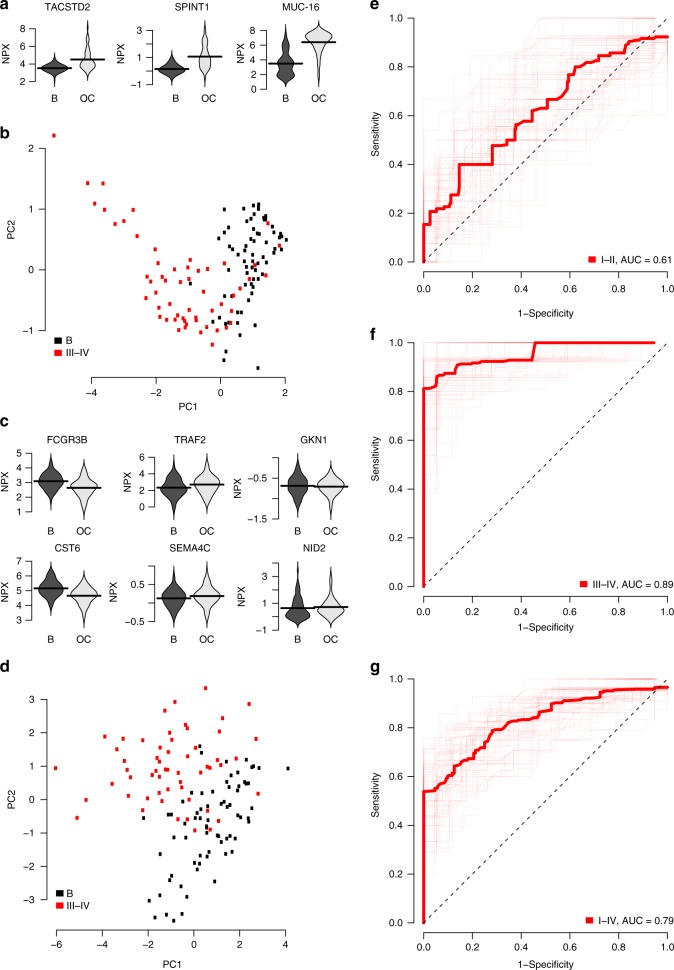
Top-ranking model performance in 1st replication cohort. **a** Distribution of protein abundance levels in NPX for the three proteins in the core in patients with Benign tumors (indicated with a ‘B’) and ovarian cancer stage III–IV (indicated with ‘OC’). Horizontal black lines indicate mean of the protein abundance levels. **b** PCA plot of the first two components using the proteins in the core. Figures show Benign tumors in black and ovarian cancer stages III–IV in red. **c** As (**a**) but for the six first additional proteins in the model. **d** As (**b**) but for the complete model with 14 proteins. **e**–**g** Receiver operating characteristic (ROC) curves of the performance of the complete model in the 1st replication cohort. From top to bottom, the ROC-curves represent Benign tumors vs. Ovarian cancer stages I–II, III–IV, and I–IV, respectively

The top-ranking of the 484 models included a three-protein core with MUCIN-16, TACSTD2, and SPINT1. This core was extended with 11 additional proteins (FCGR3B, TRAF2, GKN1, CST6, SEMA4C, NID2, CEACAM1, CLEC6A, MILR1, CA3, and CDH3). The distribution of abundance levels for the core proteins in the 1st replication in patients with ovarian cancer stages III–IV and those with benign tumors are shown in Fig. 3a. The core proteins have clearly deviating levels between the cancer cases and controls and this is further illustrated by a principal component analysis (PCA) based on the three core proteins (Fig. 3b). The additional proteins were then selected based on explained variance in the decision after adjustment for the variance explained by the proteins in the core (Methods). Therefore, some of the additional proteins (Fig. 3c) do not differ in abundance between cases and controls when examined separately, but contribute to the separation when examined in combination with the previously included proteins. The separation between benign tumors and ovarian cancer stages III–IV for the top-ranked 14-protein model is shown in the PCA in Fig. 3d.

Receiver operating characteristic (ROC) curves for benign tumors versus ovarian cancer stages I–II, III–IV, and I–IV are shown in Fig. 3e–g. Similar illustrations for the discovery and 2nd replication cohort are given as Supplementary Figs. 1 and 2. For separating benign tumors from ovarian cancer stages III–IV, the top-ranked 14-protein model had an area under the curve (AUC) of 0.9, a sensitivity = 0.99 and a specificity = 1.00 in the test-proportion of the discovery data. In the test proportion of the 1st replication data, the model had an AUC = 0.89, a positive predictive value (PPV) of 0.93, a sensitivity = 0.89 and a specificity = 0.95. This should be compared to MUCIN-16 which by itself had an AUC = 0.70, a PPV = 0.81, a sensitivity = 0.86 and a specificity = 0.85 in same cohort (Fig. 3f, Table 3). At a sensitivity above 0.93 in the 1st and 2nd replication cohorts, the model achieved a specificity of 0.27 and 0.28, respectively, and at a specificity above 0.93 a sensitivity of 0.86 and 0.80. Performance measures for the discovery and replication cohorts for all the different stages investigated are listed in Table 3.

**Table 3:** Performance of the top-ranking and the proof-of-concept model

Stage^a^	Cohort	AUC	PPV	NPV	BPse^b^	BPsp^b^	FSEse^c^	FSEsp^c^	FSPse^c^	FSPsp^c^
*Mucin-16 only*										
I–II	Discovery	0.82 (0.07)	0.68 (0.14)	0.92 (0.04)	0.85 (0.09)	0.82 (0.08)	1.00 (0.01)	0.06 (0.06)	0.60 (0.16)	0.96 (0.01)
	1st Repl.	0.51 (0.1)	0.62 (0.13)	0.79 (0.09)	0.71 (0.13)	0.71 (0.12)	1.00 (0.01)	0.20 (0.07)	0.29 (0.15)	0.94 (0.01)
	2nd Repl.	0.27 (0.15)	0.25 (0.16)	0.87 (0.09)	0.65 (0.23)	0.51 (0.22)	1.00 (0)	0.15 (0.09)	0.06 (0.12)	0.96 (0.03)
I–IV	Discovery	0.86 (0.04)	0.88 (0.08)	0.87 (0.06)	0.86 (0.06)	0.89 (0.07)	0.95 (0.01)	0.31 (0.26)	0.75 (0.11)	0.96 (0.01)
	1st Repl.	0.65 (0.08)	0.83 (0.06)	0.73 (0.10)	0.79 (0.09)	0.78 (0.08)	0.96 (0.01)	0.26 (0.12)	0.52 (0.14)	0.96 (0.02)
	2nd Repl.	0.57 (0.09)	0.78 (0.08)	0.70 (0.09)	0.69 (0.09)	0.78 (0.10)	0.95 (0.01)	0.27 (0.12)	0.45 (0.16)	0.95 (0.02)
III–IV	Discovery	0.91 (0.06)	0.95 (0.11)	0.95 (0.11)	0.96 (0.06)	0.98 (0.05)	1.00 (0)	0.06 (0.03)	0.94 (0.08)	0.96 (0.01)
	1st Repl.	0.70 (0.08)	0.81 (0.09)	0.81 (0.09)	0.86 (0.07)	0.85 (0.08)	0.98 (0.03)	0.24 (0.14)	0.68 (0.16)	0.95 (0.01)
	2nd Repl.	0.60 (0.08)	0.79 (0.10)	0.79 (0.10)	0.75 (0.09)	0.81 (0.07)	0.96 (0.03)	0.31 (0.16)	0.49 (0.14)	0.95 (0.02)
*Top-ranking*										
I–II	Discovery	0.83 (0.06)	0.74 (0.15)	0.91 (0.05)	0.81 (0.09)	0.86 (0.09)	1.00 (0.01)	0.06 (0.08)	0.60 (0.18)	0.96 (0.01)
	1st Repl.	0.61 (0.09)	0.60 (0.13)	0.75 (0.10)	0.64 (0.13)	0.70 (0.12)	0.99 (0.03)	0.04 (0.02)	0.26 (0.15)	0.95 (0.02)
	2nd Repl.	0.65 (0.18)	0.42 (0.22)	0.95 (0.05)	0.80 (0.20)	0.74 (0.17)	1.00 (0)	0.06 (0.01)	0.30 (0.27)	0.95 (0.01)
I–IV	Discovery	0.88 (0.04)	0.91 (0.06)	0.86 (0.06)	0.85 (0.06)	0.91 (0.06)	0.95 (0.01)	0.38 (0.18)	0.78 (0.09)	0.96 (0.01)
	1st Repl.	0.79 (0.06)	0.85 (0.07)	0.71 (0.09)	0.74 (0.08)	0.83 (0.09)	0.96 (0.01)	0.09 (0.14)	0.58 (0.13)	0.95 (0.02)
	2nd Repl.	0.85 (0.05)	0.88 (0.06)	0.84 (0.08)	0.86 (0.07)	0.87 (0.06)	0.95 (0.01)	0.35 (0.29)	0.73 (0.12)	0.96 (0.02)
III–IV	Discovery	0.95 (0.01)	1.00 (0.02)	1.00 (0.02)	0.99 (0.03)	1.00 (0.01)	1.00 (0)	0.04 (0)	0.99 (0.03)	0.96 (0.01)
	1st Repl.	0.89 (0.04)	0.93 (0.07)	0.93 (0.07)	0.89 (0.06)	0.95 (0.05)	0.97 (0.03)	0.27 (0.31)	0.86 (0.10)	0.95 (0.01)
	2nd Repl.	0.87 (0.05)	0.89 (0.09)	0.89 (0.09)	0.88 (0.06)	0.90 (0.08)	0.95 (0.02)	0.28 (0.31)	0.80 (0.13)	0.94 (0.01)
*Proof-of-Concept*										
I–II	Discovery	0.83 (0.06)	0.72 (0.13)	0.91 (0.05)	0.83 (0.08)	0.84 (0.08)	1.00 (0.01)	0.05 (0.06)	0.60 (0.19)	0.96 (0.01)
	1st Repl.	0.69 (0.10)	0.63 (0.11)	0.82 (0.11)	0.77 (0.13)	0.69 (0.11)	0.99 (0.02)	0.05 (0.02)	0.37 (0.15)	0.95 (0.02)
	2nd Repl.	0.70 (0.20)	0.58 (0.27)	0.95 (0.05)	0.80 (0.18)	0.82 (0.2)	1.00 (0)	0.06 (0)	0.54 (0.31)	0.94 (0.01)
I–IV	Discovery	0.88 (0.04)	0.88 (0.06)	0.89 (0.06)	0.87 (0.07)	0.90 (0.06)	0.95 (0.01)	0.40 (0.22)	0.79 (0.09)	0.96 (0.01)
	1st Repl.	0.82 (0.05)	0.87 (0.08)	0.75 (0.08)	0.79 (0.07)	0.85 (0.09)	0.96 (0.01)	0.20 (0.18)	0.66 (0.12)	0.95 (0.01)
	2nd Repl.	0.83 (0.04)	0.87 (0.07)	0.84 (0.07)	0.83 (0.08)	0.87 (0.07)	0.95 (0.01)	0.36 (0.23)	0.68 (0.11)	0.95 (0.01)
III–IV	Discovery	0.95 (0.02)	0.99 (0.03)	0.99 (0.03)	0.98 (0.04)	1.00 (0.01)	1.00 (0)	0.04 (0)	0.98 (0.04)	0.96 (0)
	1st Repl.	0.90 (0.04)	0.94 (0.06)	0.94 (0.06)	0.91 (0.07)	0.95 (0.05)	0.97 (0.03)	0.27 (0.31)	0.88 (0.10)	0.95 (0.02)
	2nd Repl.	0.84 (0.06)	0.88 (0.07)	0.88 (0.07)	0.85 (0.08)	0.89 (0.07)	0.95 (0.02)	0.32 (0.30)	0.73 (0.14)	0.95 (0.02)

^a^Performances are for benign tumors vs this stage of ovarian cancers

^b^Performances when cut-off is chosen at the best point (BP, closest point on ROC-curve to perfect classification)

^c^Performances at a point on the ROC-curves with at least 0.93 sensitivity (FSEse and FSEsp) or specificity (FSPse and FSPsp)

### Proof-of-concept model for practical use

Several factors in addition to the ability to separate cases and controls may influence the choice of the proteins included in a multiplex test, such as comparison with established tests, measurable concentration range, and sensitivity of proteins to hemolysis of red blood cells causing leakage of proteins into the plasma. Taking these limitations into account, we again started from the top-ranking core of the 484 models and allowed additional selection but restricted the search to proteins present in models with the highest performance in the discovery cohort. This list of possible additions was filtered by removing proteins sensitive to exposure to hemolysate^20^ and proteins that occur in much higher concentrations in human plasma than those in the selected core, and therefore would need to be diluted before assayed with PEA^20^. Here, we removed proteins required less than 7.5 mg/ml hemolysate, or that required dilution of 1:2025 and this filtering process retained 414 proteins. We then performed model selection as before based solely on the discovery data (benign tumors versus ovarian cancer stages III–IV) and identified a model consisted of 8 proteins. We finally added three proteins (WFDC2, KRT19, and FR-alpha) based on their previous association with ovarian cancer stages I–II in our modeling, or in the previous literature^18,21,22^. The selected 11-protein panel consisted of the three core proteins MUCIN-16, SPINT1, TACSTD2, and the additional proteins CLEC6A, ICOSLG, MSMB, PROK1, CDH3, WFDC2, KRT19, and FR-alpha. The performance of this 11-protein panel was evaluated in the two replication cohorts (Table 3). In the 1st replication cohort the AUC = 0.90, PPV = 0.94, sensitivity = 0.91 and specificity = 0.95 to distinguish benign tumors from ovarian cancer stage III–IV.

### Validation of proof-of-concept model

**Fig. 4:**
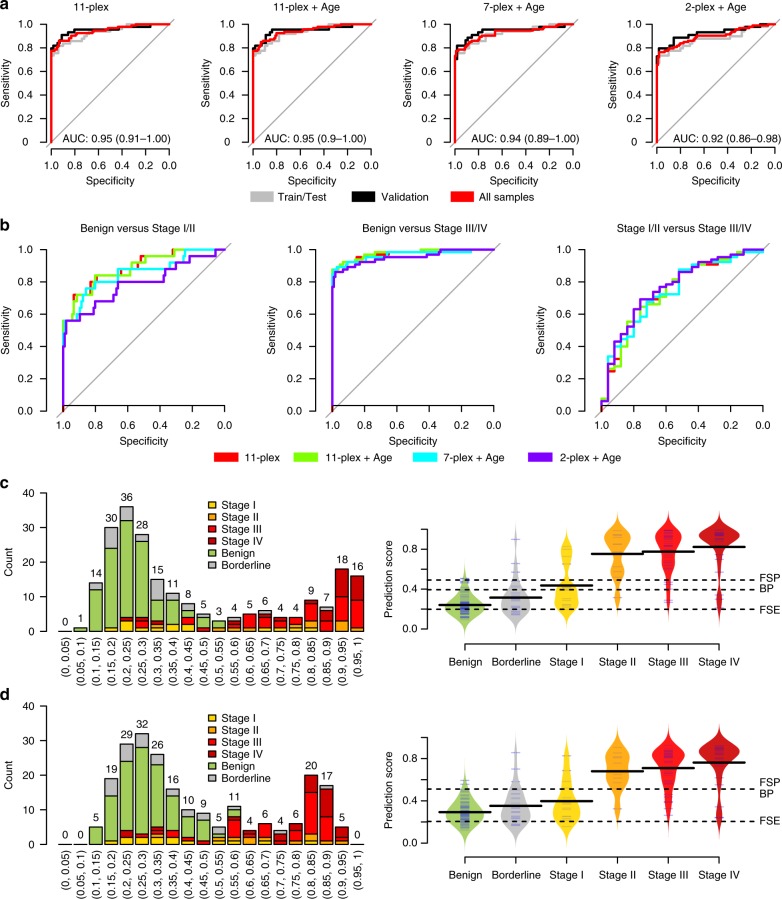
Final models’ performance in the 3rd replication cohort. **a** ROC-curves for the test/training (gray), validation (black) and final model (red) for each of the 4 models. The AUC is taken from the performance in the validation partition. All models were trained on benign vs malign (stages I–IV) samples. **b** ROC-curves for the 4 final models when evaluated on subsets of stages. **c** Distribution of outcomes in ranges of prediction scores (left) for the ‘11-plex + Age’ model and distribution of prediction scores for each outcome (right). In the right panel, the three cut-offs for ‘best-point (BP)’, ‘focus on sensitivity (FSE, sensitivity ≥0.98)’ and ‘focus on specificity (FSP, specificity ≥0.98)’ are illustrated by horizontal dashed lines. The solid black lines indicate the mean prediction score in each outcome group. **d** As (**c**) but for the ‘2-plex + Age’ model

In order to validate the performance of the 11-protein proof-of-concept model we then developed a custom PEA-assay^23^ that measured the 11 proteins and used this to characterize protein abundance levels in a third replication cohort (Tables 1 and  3). Here, calibration samples (see Methods for details) were included in the custom assay in order to have the final readout in absolute protein concentrations rather than NPX. Concentration ranges of the custom assay and performance measures are given in Supplementary Data 4. The third replication cohort was first split into two equal parts, a training set, and a validation set, in terms of size and proportion of benign and malignant (stages I–IV) tumors. A linear regression model was then trained, employing fivefold cross-validation using the training part only. In the training-set this model achieved an AUC of 0.93 (%95 CI 0.88–0.98) in separating benign from stages I–IV (malignant), and a similar performance was observed in the validation set (AUC = 0.95, %95 CI 0.91–1.00, Fig. 4a). Since the performance in the validation set was highly similar to the training set with no statistical difference (DeLong's test, *p*-value = 0.53), a final model was generated using fivefold cross-validation with the entire third replication cohort in order to capture as much variation as possible. This model (Supplementary Data 4) achieved an AUC of 0.94 (%95 CI 0.91–0.98) with a sensitivity of 0.86 at a specificity of 0.93 at the point closest to perfect classification (Supplementary Data 5). Next, we trained a model using the 11 proteins and age at diagnosis (Supplementary Data 4, Fig. 4a). As before, there was no difference in AUC for the training and validation sets (DeLong's test, *p*-value = 0.62) and using the whole cohort, this model achieved an AUC of 0.94 (%95 CI 0.91–0.98) with a sensitivity of 0.85 at a specificity of 0.93 at the point closest to perfect classification. This was determined at a cut-off of 0.3937. We also recorded cut-offs for focus on sensitivity or specificity over 0.98. With this focus, the model achieved sensitivity and specificity of 0.99/0.31 or 0.77/0.98 at cut-offs of 0.2501 and 0.5474, respectively (Table 4). We also trained a model based on WFDC2, Mucin-16 and age at diagnose for comparison and a model based on age and 7 biomarkers (MUCIN-16, TACSTD2, MSMB, PROK1, WFDC2, KRT19, and FR-alpha) that excluded the proteins with the highest technical variation in our custom-assay (Supplementary Fig. 3). In both these models there was no difference in performance between the training and validation proportions of the data (DeLong's test *p*-values = 0.60 and 0.34, respectively) and again, final models were created based on the entire cohort. Performance measures for all 4 models based on the custom assay are available in Supplementary Data 5. In general, the models trained on benign vs malignant (stages I–IV) tumors are better at separating late stages (stages III–IV, AUC-range 0.95–0.98) than early (stages I–II, AUC-range 0.79–0.88) and has lower performance separating stages I–II from stages III–IV (AUC-range 0.74–0.77, Fig. 4b, Supplementary Data 5).

**Table 4:** Performance of the custom assay in the independent replication cohort, combined analysis

	Full model^a^				MUCIN-16 and WFDC2 and Age			
	Benign vs Malign	Benign vs Stage I–II	Benign vs Stage III–IV	Stage I–II vs Stage III–IV	Benign vs Malign	Benign vs Stage I–II	Benign vs Stage III–IV	Stage I–II vs Stage III–IV
AUC	0.94 (0.91-0.98)	0.88 (0.81–0.96)	0.98 (0.96-1)	0.74 (0.63–0.86)	0.90 (0.85–0.95)	0.79 (0.67–0.91)	0.95 (0.92–0.99)	0.77 (0.65–0.88)
PPV^b^	0.92	0.71	0.90	0.78	0.97	0.88	0.97	0.80
NPV^b^	0.88	0.93	0.95	0.62	0.83	0.90	0.92	0.55
BPcut^c^	0.3937	0.3937	0.3937	0.3937	0.5117	0.5117	0.5117	0.5117
BPse	0.85 (0.76–0.91)	0.68 (0.48–0.84)	0.92 (0.85–0.98)	0.92 (0.86–0.98)	0.76 (0.67–0.85)	0.56 (0.36–0.76)	0.86 (0.77–0.94)	0.86 (0.77–0.94)
BPsp	0.93 (0.88–0.98)	0.93 (0.88–0.98)	0.93 (0.89–0.97)	0.32 (0.16–0.52)	0.98 (0.95–1.00)	0.98 (0.95–1.00)	0.98 (0.95–1.00)	0.44 (0.24–0.64)
FSEcut^c^	0.1976	0.1976	0.1976	0.1976	0.2047	0.2047	0.2047	0.2047
FSEse	0.99 (0.96–1.00)	1.00 (1.00–1.00)	1.00 (1.00–1.00)	1.00 (1.00–1.00)	0.99 (0.97–1.00)	0.96 (0.88–1.00)	1.00 (1.00–1.00)	1.00 (1.00–1.00)
FSEsp	0.31 (0.23–0.40)	0.31 (0.23–0.41)	0.31 (0.23–0.40)	0 (0.00–0.00)	0.19 (0.11–0.26)	0.19 (0.12–0.26)	0.19 (0.11–0.27)	0.04 (0.00–0.12)
FSPcut^c^	0.4908	0.4908	0.4908	0.4908	0.5117	0.5117	0.5117	0.5117
FSPse	0.77 (0.69–0.86)	0.56 (0.36–0.76)	0.88 (0.80–0.95)	0.88 (0.78–0.95)	0.76 (0.68–0.85)	0.56 (0.36–0.76)	0.86 (0.77–0.94)	0.86 (0.77–0.94)
FSPsp	0.98 (0.95–1.00)	0.98 (0.95–1.00)	0.9 (0.95–1.00)	0.44 (0.24–0.64)	0.98 (0.95–1.00)	0.98 (0.95–1.00)	0.98 (0.95–1.00)	0.44 (0.24–0.64)

^a^Proof-of-concept model plus age

^b^Performances when cut-off is chosen at the best point (BP, closest point on ROC-curve to perfect classification)

^c^Cut-off thresholds calculated in the Benign vs. Malign models and applied to difference subgroups. The BPcut is taken at the point on the ROC-curve closest to perfect performance. The FSEcut is taken from point with highest specificity when requiring at least 0.98 sensitivity. The FSPcut is taken from point with highest sensitivity when requiring at least 0.98 specificity. All cells: numbers in parentheses represent 95% confidence intervals

Finally, we included also samples from the third replication cohort that had been diagnosed with borderline ovarian cancer and plotted the prediction scores from the 11 proteins plus age model alongside of the benign and malignant samples (Fig. 4c). From Fig. 4c left panel it is clear that only samples with stages II or higher have prediction scores above 0.9 while only benign or borderline samples have a score lower than 0.15. As compared to the use of the WFDC2, MUCIN-16 plus age model (Fig. 4d, left panel), there is a more than 2-fold increase in the number of women that fall in these prediction score categories, i.e., above 0.9 (*n* = 34 vs 5) or below 0.15 (*n* = 15 vs 5). This is also illustrated in Figure 4c, d, right panels, where the distribution of prediction scores for each diagnosis is shown. The cut-offs used for “best point”, high sensitivity or high specificity are also illustrated by horizontal lines. The prediction scores from the 11 protein plus age model in late stage ovarian cancers (Stages IV) are significantly higher than that of the 2 protein plus age model, while the predictions scores in the Benign group are significantly lower (Wilcox-test, Bonferroni adjusted cut-off: 0.05/6 = 8.3 × 10^–3^, *p*-values = 5.5 × 10^−3^ (Stage IV) and 2.0 × 10^−6^ (Benign), respectively). The prediction scores for the borderline samples fall between the benign and stage I samples (Fig. 4c, right panel) and there is not obvious cut-off that for distinguishing these from either the benign or malignant tumor samples.

## Discussion

The current study was designed to identify mutually exclusive predictive biomarker signatures containing up to 20 plasma proteins differentiating benign tumors from different stages of ovarian cancers. We started from a large number of plasma proteins, not selected based on prior association with ovarian cancer, utilizing high-throughput multiplexed proteomics assays. The models were developed using a discovery cohort, and the performance of the models was then evaluated using two replication cohorts. In addition to the 484 biomarker signatures obtained using our computerized strategy, we developed one model considering protein-specific criteria such as abundance range and sensitivity to hemolysis. Finding combinations of predictive, robust, biomarkers is computationally intensive, and with many hundreds of proteins, exhaustive searches of combinations of up to 20 proteins is not feasible. To this end, we developed a strategy for identification of highly predictive unique signatures using hierarchical exclusion of individual proteins. By design, this led to the discovery of many signatures that did not contain MUCIN-16, although this protein was the strongest univariate biomarker among the ones we studied. Overall, the signatures without MUCIN-16 contained a higher number of different proteins than signatures with MUCIN-16, but there was no clear difference in prediction performance of the group with and without MUCIN-16. Our top-ranking model achieved a sensitivity of 0.99 and specificity of 1.0 in the test proportion of the discovery data for separating benign tumors from ovarian cancer stage III–IV. A recent study by Boylan and colleagues^18^ reports perfect classification (AUC = 1.0 and AUC = 1.0) of benign tumors and late-stage ovarian cancer and very high performances (AUC = 0.98 and AUC = 0.85) using either MUCIN-16 or WFDC2 alone, by analysis of a single cohort with proteins measured using the same PEA technology as in our study. In our 1st replication cohort, MUCIN-16 alone had lower AUCs of 0.70, 0.65, and 0.51 for separating benign tumors from ovarian cancer stages III–IV, I–IV, and I–II, respectively (Fig. 3f, g). The difference in performance between our study and that by Boylan and colleagues^18^ could be due to geographic origin of the cohorts (USA and Sweden), biological nature of the sample (i.e., serum versus plasma), or differences in sample sizes and model evaluations. Boylan and colleagues^18^ used 21 women with benign conditions and 21 with late-stage ovarian cancer, as compared to 71 and 56 in our study. Another study by Han and colleagues^16^ reported a sensitivity of 0.87 at a specificity of 1.0 for separating benign tumors from ovarian cancer stage I–IV, using the four proteins MUCIN-16, E-CAD, WFDC2, and IL-6. Our top-ranked model had a sensitivity of 0.85 and specificity of 0.91 under the same conditions. Similar to the results of these previous studies^16,18^, the performance of our models in the test-proportion of the discovery data is very good, with some models showing perfect classification. We also evaluated the selected models in two replication cohorts and found the performance similar, while somewhat lower than in the discovery set. This either implies that there are underlying differences between the cohorts, such as in pre-analytical conditions, or that the models are over-trained with respect to the samples in the discovery cohort. The performance in the test-proportion of the discovery cohort should, therefore, be considered less certain than the results obtained in the replication cohorts. In our study, the benign tumors and the cancer samples from the 2nd replication cohort differ in pre-analytical context, which could explain part of the lower performance as compared to using the 1st replication cohort. We then implemented our final, proof-of-concept, model into a custom assay reporting in absolute protein concentrations. Since the readout for the custom assay differ from the standard PEA-readout used in the discovery and first two replication cohorts the model coefficients needed to be retrained. This could lead to overfitting of the model to the investigated cohort. To try and overcome this, we employed a 5-fold cross-validation schema to train the final model using 50% of the third replication cohort and kept a withheld validation proportion to estimate the performance on samples not used in the training. As the performance in these two subsets did not essentially differ, the retraining of the models does not seem to be overfitted with respect to the samples used. We, therefore, used a 5-fold cross-validation schema using the entire third replication cohort for the final model fitting. This does however not necessarily guarantee that the performance of the model will remain the same in additional cohorts with e.g., different ethnic compositions or when applied to samples with large differences in pre-analytical handling. We also noted that the performance of our model is slightly better in the third replication cohort, where the AUC was 0.95 (%95 CI 0.91–1.00) in the validation-proportion, as compared to the performance in the test-proportion of the discovery and first two replication cohorts, where the model had AUCs ranging from 0.82–0.88. This could be due to the wider dynamic range of the custom assay, but indicates that the performance of the model is robust. A second contributing factor could be that cases are compared to a group diagnosed with benign tumors, representing heterogenous conditions. In the third replication cohort used here, the most common benign tumors were diagnosed as ‘Serous cyst’ (29.2%), followed by ‘Mucinous cyst’ (20.8%), ‘Mesonephric or inclusion cyst’ (17.0%), ‘Stromal cyst’ (11.3%), ‘Teratoma’ (11.3%), ‘Endometrioma’ (6.6%) and finally, ‘Myoma’ (3.8%). This highlights the importance of understanding the context in which a biomarker test is to be used as compared to the setting used for development of the model.

We note that there is a group (*n* = 3) of late=stage cancers that gets low prediction scores using our model (Fig. 4c, right panel, stage IV). Comparing this group with low scores to the others diagnosed with the same stage (*n* = 21), we found no statistical difference (wilcox.test, two-sided) in either age (*p* = 0.50), BMI (*p* = 0.93). These 3 samples do however have lower clinical Mucin-16 values (*p* = 0.011) with a median value of 49.5 U/ml compared to the group with high prediction scores that have a median value of 1312.5 U/ml. From this observation, it is clear that there are samples that will still be hard to find or distinguish using the biomarker model presented here.

Some of the proteins in the 11-protein panel, aside from MUCIN-16 and WFDC2 (HE4), have previously been associated to ovarian cancer. *TACSTD2* (tumor-associated calcium signal transducer 2) expression has been associated with decreased survival of ovarian cancer and proposed as a prognostic factor^24^, and a biomarker for targeted therapy^25^. SPINT1 (matriptase, HAI-2) is a type II transmembrane serine protease expressed on epithelial ovarian tumor cells. In advanced stage ovarian tumors, matriptase is expressed in the absence of HAI-1, its inhibitor, indicating that an imbalance between matriptase and HAI-1 is important in the development of ovarian disease^26^. Matriptase has also been proposed as an adjuvant therapeutic target for inhibiting ovarian cancer metastasis^27^. Analysis of circulating tumor cell RNA has seen an increased expression of *KRT19* (keratin, type I cytoskeletal 19), but no studies of the plasma protein level have been performed^28^. FR-alpha (folate receptor alpha, FR-alpha) is a GPI-anchored glycoprotein and serum levels has been found to be elevated in ovarian cancer patients^29,30^ and correlated to both clinical stage and histological type^31,32^. Finally, decreased expression of *MSMB* (beta-microseminoprotein) has been shown to correlate with reduced survival of invasive ovarian cancer^33^.

In order to study the potential of using the protein panels in diagnosis or screening, we determined their performance after tuning the models prioritizing either specificity or sensitivity. A diagnostic test for women with a TVU indication of adnexal ovarian mass must possess a high sensitivity, but can accept a moderate specificity. Previous studies predicting the risk of malignancy in adnexal ovarian mass using TVU only^5^, reports sensitivities ranging from 99.7 to 89.0% with specificities of 33.7 to 84.7% for calculated risk scores of 1 to 30% and positive predictive values ranging from 44.8 to 75.4%. At a minimum sensitivity of 0.98 our final 11-plex + Age model distinguishes between women with benign tumors and ovarian cancer stage I–IV with a specificity of 0.31 (%95 CI 0.23–0.40) at a sensitivity of 1.0 and positive and negative predictive values of 0.47 and 1.00, respectively. An earlier report^34^ retrospectively examined the predictive value of MUCIN-16 and WFDC2 among Swedish women that underwent surgery with suspected ovarian cancer. Out or 373 women, 58% were found to have benign tumors and 30% have ovarian cancer (15% stage I–II, 15% stage I–IV). That study reported a sensitivity of 61.9% at specificity of 75% with a positive predictive value of 31.3% for MUCIN-16 and WFDC2 combined. Thus, the performance measures of the model presented here are higher than the current clinically used biomarker combinations, but lower than the highest reported performances of clinical specialists, albeit with a higher positive predictive value. A combined use of both TVU and a biomarker test is likely to give even higher specificity. An indication of the potential for using the protein model for identification of women at risk in population screening was obtained by studying the sensitivity at high specificity. At a minimum specificity of 0.98, the final protein panel has sensitivity of 0.77 (%95 CI 0.69–0.86) in distinguishing benign tumors from women with ovarian cancer stages I–IV (Table 4). Further studies are needed using samples collected at different time-points prior to diagnosis to evaluate the potential of using the panel in population screening. In screening, the aim is not to distinguish between benign tumors and cancer, but between healthy women and cancer, and it is likely that there will be more pronounced differences when comparing to a healthy population. In support of this notion, we have shown in a previous study^19^ that the sensitivity to distinguish population controls from stage I–IV cancer was 0.62 and stage III–IV was 0.78. Future studies including age-matched population controls have to be conducted to determine the performance of the final 11-protein biomarker set in population screening.

In summary, we have developed a strategy for the identification of protein cores that resulted in mutually exclusive combinations of protein signatures that can separate between benign tumors and ovarian cancers. The results demonstrate the ability to achieve high performance characteristics without including MUCIN-16. We also show that broad searches for novel combinations of protein biomarkers that on their own are not necessarily good predictors is a powerful approach for finding relevant biomarkers for disease.

## Methods

### Samples

Plasma samples of women with benign and malignant ovarian tumors were collected from either the U-CAN collection^35^ at Uppsala Biobank, Uppsala University, Sweden or the Gynaecology tumor biobank^36^ at Sahlgrenska University Hospital, Göteborg, Sweden, as previously described^19^ (Table 1). All samples from the biobanks were included based on ovarian cancer diagnosis or patients that had been surgically diagnosed with benign conditions based on suspicion of ovarian cancer. Since the distribution of individual protein abundance levels in the samples was unknown and all available samples were to be extracted from the biobanks, no univariate power calculations were carried out prior sample collection. Patients that had received neoadjuvant treatment prior to surgery were excluded from the analysis. The samples from U-CAN have been deposited from 2010 to 2016. The samples in the Gynaecology tumor biobank were deposited from 2001 to 2018 with the discovery cohort samples collected in 2001–2010, the first replication cohort 2012–2015 and the third replication cohort in 2016–2018. All tumors were examined by pathologist specialized in gynaecologic cancers for histology, grade, and stage according to International Federation of Gynaecology and Obstetrics (FIGO) standards. All plasma samples were frozen and stored at −70 °C. The discovery cohort consisted of 90 patients diagnosed with benign tumors and 79 patients with ovarian cancer stages I–IV. Samples were collected at time for primary surgery under full anesthesia but before incision. All women had at least 6 h fasting before sample collection. The first replication cohort consisted of 71 patients diagnosed with benign tumors and 100 patients with ovarian cancer stages I–IV and were collected under the same conditions as the discovery cohort. The second replication cohort consisted of 77 patients with ovarian cancer stages I–IV. The second replication samples were collected at time of diagnosis, from awake patients, by a trained nurse. The third replication cohort consisted of 106 patients with benign conditions, 28 with borderline diagnosis and 93 with ovarian cancer stages I–IV. All samples from the third replication cohort were collected at time of diagnosis, from awake patients, by a trained nurse.

### Ethical compliance

Written consent was obtained from all participants before the samples used here were deposited in the biobanks. One woman with her sample in the Gynaecology tumor biobank at Sahlgrenska University Hospital withdrew her consent after the samples had been acquired from the biobank and this sample was excluded from the analyses conducted here. The study was approved by the Regional Ethics Committee in Uppsala (Dnr: 2016/145) and Göteborg (Dnr: 201-15).

### Protein measurements

We have previously quantified 460 proteins from the Olink Multiplex Cardiovascular II, Cardiovascular III, Inflammation, Neurology and Oncology panels in the discovery cohort using the proximity extension assay (PEA)^19^. The PEA is an affinity-based assay which characterizes abundance levels of a pre-determined sets of proteins. For each measured protein, a pair of oligonucleotide-labeled antibodies probes target the protein and if both probes are in close proximity, a PCR target sequence is formed by a proximity-dependent DNA polymerization event. The resulting sequence is then detected and quantified using standard real-time PCR. Forty-two of these have also been quantified in the first and second replication cohorts using PEA in two custom-design 21-plex panels^19,23^. The 42 proteins in the custom-design 21-plex proteins were selected from the 460 based on their relationship with gynecological cancers as described earlier^19^. Here, an additional 552 proteins were analyzed using 6 additional PEA-panels (the Olink Multiplex Cardiometabolic, Cell Regulation, Development, Immune Response, Metabolism and Organ Damage) and real-time PCR using the Fluidigm BioMark™ HD real-time PCR platform^37^ in the discovery and replication cohorts. A complete list of the 1012 assays corresponding to 981 unique proteins are listed in Supplementary Data 1. The samples were randomized across plates and normalized for any plate effects using the built-in inter-plate controls according to manufacturers’ recommendations. The PEA gives abundance levels in NPX (Normalized Protein eXpression) that is on log2-scale. Each assay has an experimentally determined lower limit of detection (LOD) defined as three standard deviation above noise level. Here, all assay values below LOD were replaced with the defined LOD-value. Samples and proteins that did not pass the quality control were removed. After quality control, 42 proteins from the custom panels and 551 from the additional 6 panels were kept. Assay characteristics including detection limits calculations, assay performance and validations are available from the manufacturer (www.olink.com).

The proteins from the proof-of-concept model were quantified using a custom 11-plex assay in the analysis of the third replication cohort. Description of the development process for combining protein assays into custom multiplexed reactions and the technology behind having final readout in absolute concentrations have been published earlier in a white-paper^23^. In brief, normal and disease state occurring protein concentration ranges in circulating plasma are accounted for and the dynamic range for each individual assay optimized to take this into account. In addition, standard curves for all individual proteins have to be established by analysis of a wide range of recombinant antigen concentrations. In the final test, triplicate measures of calibrators at 4 known concentrations (blank, low, mid and high concentrations) of each protein were included in each run. These were used for normalization and the normal PEA-readout (NPX) and to estimate absolute concentrations by comparing to established standard curves. Here, each sample in the third replication cohort was run in duplicates or triplicates and a mean value over the replicates was used in the analyses. If all readouts were below or above the limits of detections, no mean-value was calculated but instead replaced with the LOD-values as described above. The data were then transformed to log2-scale.

### Model generation

In order to identify non-overlapping models, we devised a two-stage strategy. In brief, the strategy first identifies a small set of proteins, a ‘core’, typically consisting of 2–6 proteins with relatively high performance. This core is then extended with additional proteins creating a full model of up to 20 proteins. The whole process is then repeated excluding one protein at a time from the core ensuring that the “next” core does not overlap with a previously detected core. In detail, the strategy breaks down into the following steps. First, the discovery set was randomly split into a training and a test set with 50% of the samples in each. A linear regression model was generated on the training set using the R-package ‘glmnet’^38^ with ‘alpha’ = 0.9 and optimized using 10-fold cross-validation in the training-set as implemented by the ‘cv.glmnet’-function. The training/test-split was then repeated 50 times and a ‘core’ was then defined as consisting of the proteins present in at least 70% of the generated models from the 50 runs. In order to find mutually exclusive cores, the core-generating process was repeated in a recursive manner, excluding one protein at a time from the previous core from the available protein pool presented to ‘cv.glmnet’. For each newly generated core, the process was then repeated unless the core contained more than a specific number of proteins or had a sensitivity or specificity below a specified cut-off. For each new search, all previously excluded proteins were made unavailable to the current selection. For computational reasons, the searches were cancelled if more than 20 proteins had been excluded. The process of defining the cores is outlined in Fig. 1a, b. In the second step of the strategy, each core was extended to a full model by adding additional proteins. These additional proteins were selected from the pool of available proteins in a stepwise forward selection. First, the variance in the decision explained by the core was removed by keeping the residuals from a linear model generated with the protein values in the core as input and the decision as output. Then, the variance explained by any other available protein in this adjusted outcome was calculated and the protein explaining the most remaining variance in the decision was added to the model and the contribution of that protein to the explained variance in the decision was adjusted for as described above. The addition of proteins was repeated until the best candidate protein did not explain more than 1% of remaining variance or the total number of proteins in the model exceeded 20 proteins (Fig. 1c). Different parameter settings were used to identify models that could have either high sensitivity or high specificity or both, depending on the final application and to account for the fact that it is much more difficult to separate stages I and II from benign tumors compared to stages III and IV. For benign tumors versus stages I–II, the core had to be 2–6 proteins in length and have a sensitivity of at least 0.8, or a sensitivity and specificity above 0.6. For stages III–IV, the allowed core size was 2–5, and had to have a sensitivity above 0.8 or a sensitivity and specificity above 0.7. Finally, for stages I–IV the allowed core size was 2–6 proteins, and the models were required to have a sensitivity above 0.8 or a sensitivity and specificity above 0.7.

### Performance estimates in the first two replication cohorts

As the second replication cohorts lacked patients with benign tumors, the benign tumors from the first replication cohort was used in both replication cohorts. Due to the relativeness of the NPX-scale and that the data in the discovery and replication sets were generated in different laboratory analysis runs, including parts of the data that were generated using a custom-panel^19^, the replication cohorts were split into a test and training set (50–50) and model coefficients were re-determined with the ‘lm’ function in R. The performance of the models was then estimated in the test proportion of each replication cohort separately. This was repeated 50 times for each model and the mean and standard deviation of sensitivity, specificity, positive and negative predictive values (PPV/NPV) and AUCs were recorded. PPV was calculated as TP/(TP + FP) where TP is the number of true positives and FP the number of false positives. NPV was calculated as TN/(TN + FN) where TN is the number of true negatives and FN is the number of false negatives. The sensitivity and specificity were calculated at three different points on the ROC curve. The ‘best point,’ defined as the closest (Euclidean distance) point to perfect classification, and by selecting a minimum sensitivity or specificity of 0.93.

### Fixation of the models in absolute concentration

The third replication cohort and was first split into two equal parts, a training set and a validation set, in terms of size and proportion of benign and malign (stages I–IV) samples. A linear regression model was then trained employing fivefold cross-validation using the training part only. The models were trained using the ‘cv.glmnet’ with alpha = 0. The performance of the model was then evaluated on the validation set. Difference in performance (AUC) from the training and validation was evaluated by a DeLong-test as implemented in the R-package ‘pRoc’^39^. When no difference in performance was detected between the training and validation sets, a final model was generated fivefold cross-validation as above using all samples with benign or malign (stages I–IV) status. Model coefficients was extracted from the cv-stage at a λ within one standard error of the minimum (‘lambda.1se’ in ‘cv.glmnet’). R-package ‘caret’^40^ was used to train the final model. In the modeling, the raw output from the linear regression was transformed to lie between 0 and 1 by a link-function, $f\left( x \right) = \frac{{e^x}}{{(e^x + 1)}}$.

### Statistics and reproducibility

All calculations were done using R^41^ (version 3.4.2). Models were fitted using functions from the R-package ‘glmnet’^38^ (version 2.0–16), ‘caret’^40^ (version 6.0–80) and ‘pROC’^39^ (version 1.12.1). Performance measures for the PEA-panels are available from the manufacturer’s webpage, www.olink.com. A full description of the custom PEA assay including assay performance measures, concentration ranges of all ingoing variables, all model coefficients, cut-offs, and prediction performance measures are given in Supplementary Data 4 and 5.

### Reporting summary

Further information on research design is available in the Nature Research Reporting Summary linked to this article.

## Supplementary information

Description of Additional Supplementary Files

Supplementary Information

Reporting Summary

Supplementary Data 1–5

## Data Availability

The complete data including replicate measurements in absolute concentrations for the 11-plex used to validated the performance of the final model is publicly available via Figshare at 10.6084/m9.figshare.7642268.
