# Supplementary material for: High throughput proteomics identifies a high-accuracy 11 plasma protein biomarker signature for ovarian cancer
Source: Commun Biol. 2019 Jun 20;2:221. doi: 10.1038/s42003-019-0464-9 (PMC6586828; doi:10.1038/s42003-019-0464-9)
Supplement: Supplementary file 1 — Description of Additional Supplementary Files [file 42003_2019_464_MOESM1_ESM.docx]

**Supplementary Data Legends**

**Supplementary Data 1**

ID and description of all proteins assayed. Includes genomic location for the gene encoding the proteins with coordinates in Hg19.

**Supplementary Data 2**

Performance measures (AUC, PPV, NPV, sensitivities and specificities for all 484 models in the discovery and first 2 replication cohorts.

**Supplementary Data 3**

Summery statistics of comparisons of performances of models with or without Mucin-16 (CA125)

**Supplementary Data 4**

Performance measures (Inter and Intra %CV), unit and ranges of the custom assay and model coefficients for calculations of final scores.

**Supplementary Data 5**

Performance measures (AUC, PPV, NPV, sensitivities and specificities) for the models based on the custom assay in the 3^rd^ replication cohort.
