# Supplementary material for: High throughput proteomics identifies a high-accuracy 11 plasma protein biomarker signature for ovarian cancer
Source: Commun Biol. 2019 Jun 20;2:221. doi: 10.1038/s42003-019-0464-9 (PMC6586828; doi:10.1038/s42003-019-0464-9)
Supplement: Supplementary file 2 — Supplementary Information [file 42003_2019_464_MOESM2_ESM.pdf]

Supplementary Figures

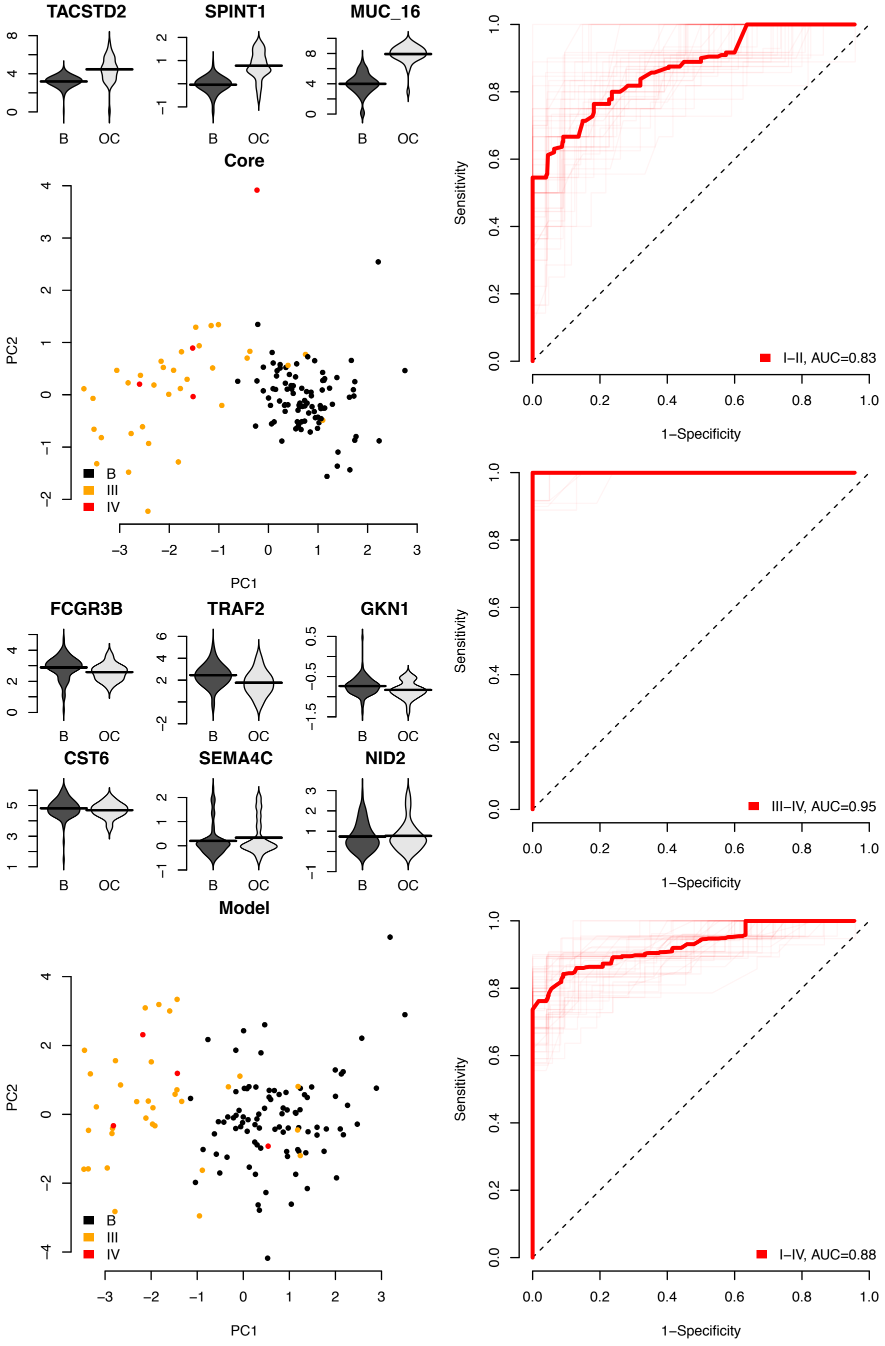

Supplementary Figure 1. **Top-ranking model performance in discovery cohort. (A)** Distribution of protein abundance levels in NPX for the three proteins in the core in patients with Benign tumours (indicated with a 'B') and Ovarian Cancer stage III-IV (indicated with 'OC'). Horizontal black lines indicate mean of the protein abundance levels. **(B)** PCA plot of the first two components using the proteins in the core. Figures shows Benign tumours in black and Ovarian Cancer stage III-IV in orange and red. **(C)** As (A) but for the six first additional proteins in the model. **(D)** As (B) but for the complete model with 14 proteins. **(E-G)** Receiver Operating Characteristic (ROC) curves of the performance of the complete model in the discovery cohort. From top to bottom, the ROC-curves represent Benign tumours vs. Ovarian cancer stages I-II, III-IV and I-IV respectively.

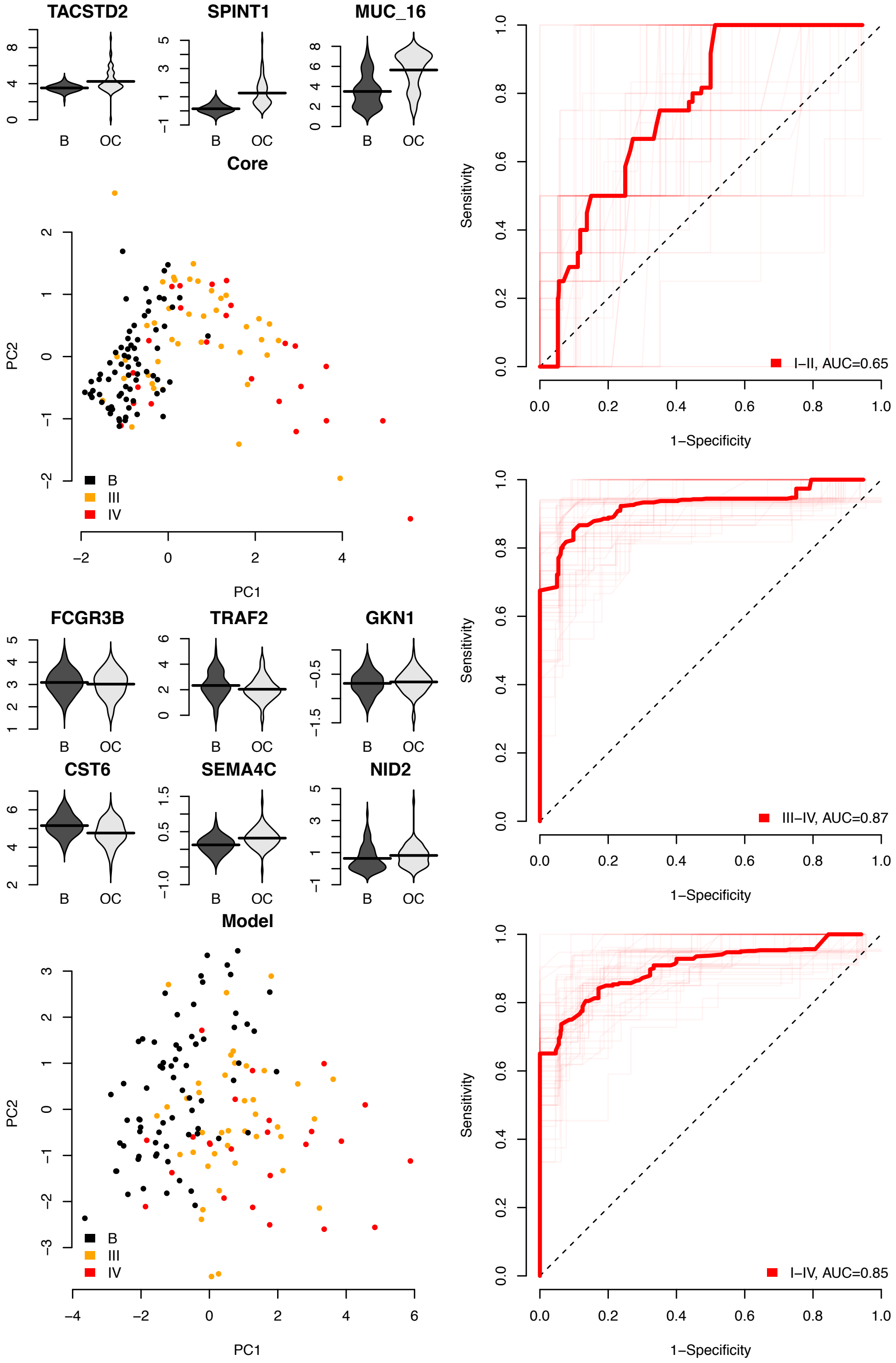

Supplementary Figure 2. **Top-ranking model performance in the 2nd replication cohort. (A)** Distribution of protein abundance levels in NPX for the three proteins in the core in patients with Benign tumours (indicated with a 'B') and Ovarian Cancer stage III-IV (indicated with 'OC'). Horizontal black lines indicate mean of the protein abundance levels. **(B)** PCA plot of the first two components using the proteins in the core. Figures shows Benign tumours in black and Ovarian Cancer stage III-IV in orange and red. **(C)** As (A) but for the six first additional proteins in the model. **(D)** As (B) but for the complete model with 14 proteins. **(E-G)** Receiver Operating Characteristic (ROC) curves of the performance of the complete model in the 2nd replication cohort. From top to bottom, the ROC-curves represent Benign tumours vs. Ovarian cancer stages I-II, III-IV and I-IV respectively.

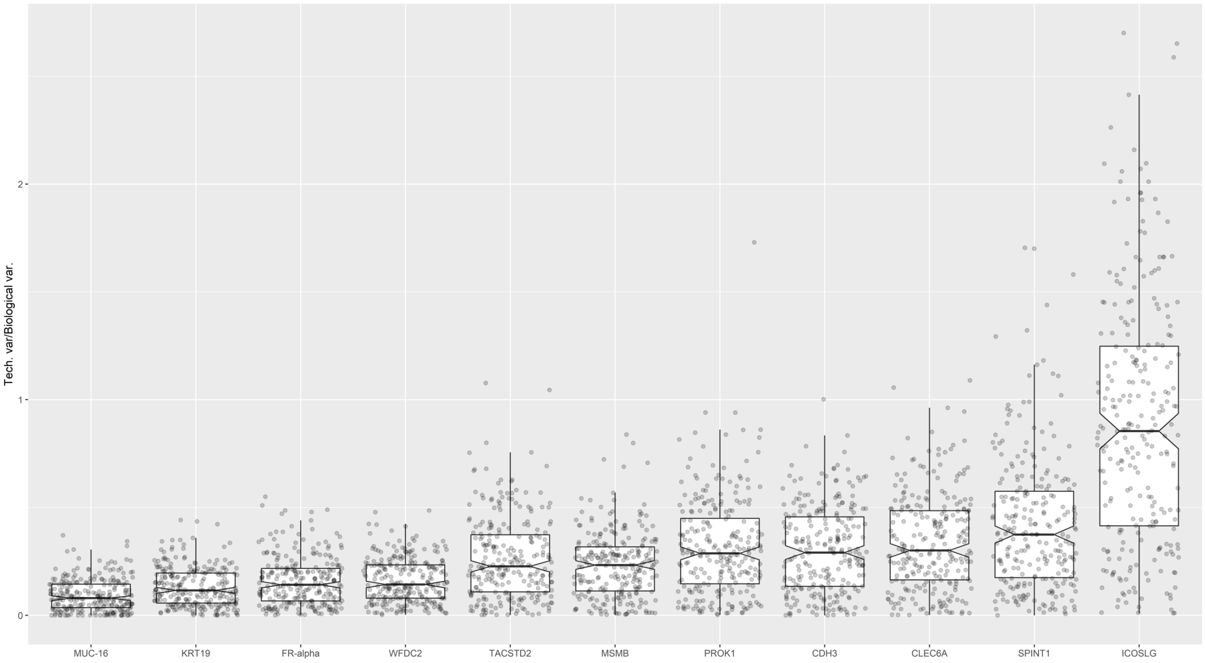

Supplementary Figure 3. **Between run variation for custom assays.** The ratio (y-axis) between technical and biological variation is shown for each assay. The points indicate subjects. The assays are sorted on median ratio from low (best) to high (worst). For each assay, technical variation was estimated as the maximum concentration difference between replicates for each subject. Biological variation was estimated as the standard deviation calculated on mean concentration for all subjects and assays. Log2 concentrations was used for the calculations.
