# Supplementary material for: High throughput proteomics identifies a high-accuracy 11 plasma protein biomarker signature for ovarian cancer
Source: Commun Biol. 2019 Jun 20;2:221. doi: 10.1038/s42003-019-0464-9 (PMC6586828; doi:10.1038/s42003-019-0464-9)
Supplement: Supplementary file 3 — Reporting Summary [file 42003_2019_464_MOESM3_ESM.pdf]

nature research

Corresponding author(s): Ulf Gyllensten

Last updated by author(s): Feb 13, 2019

Reporting Summary

Nature Research wishes to improve the reproducibility of the work that we publish. This form provides structure for consistency and transparency in reporting. For further information on Nature Research policies, see Authors & Referees and the Editorial Policy Checklist.

Statistics

For all statistical analyses, confirm that the following items are present in the figure legend, table legend, main text, or Methods section.

n/a	Confirmed	
☐	☒	The exact sample size (*n*) for each experimental group/condition, given as a discrete number and unit of measurement
☐	☒	A statement on whether measurements were taken from distinct samples or whether the same sample was measured repeatedly
☐	☒	The statistical test(s) used AND whether they are one- or two-sided<br>*Only common tests should be described solely by name; describe more complex techniques in the Methods section.*
☐	☒	A description of all covariates tested
☐	☒	A description of any assumptions or corrections, such as tests of normality and adjustment for multiple comparisons
☐	☒	A full description of the statistical parameters including central tendency (e.g. means) or other basic estimates (e.g. regression coefficient) AND variation (e.g. standard deviation) or associated estimates of uncertainty (e.g. confidence intervals)
☒	☐	For null hypothesis testing, the test statistic (e.g. *F*, *t*, *r*) with confidence intervals, effect sizes, degrees of freedom and *P* value noted<br>*Give P values as exact values whenever suitable.*
☒	☐	For Bayesian analysis, information on the choice of priors and Markov chain Monte Carlo settings
☒	☐	For hierarchical and complex designs, identification of the appropriate level for tests and full reporting of outcomes
☒	☐	Estimates of effect sizes (e.g. Cohen's *d*, Pearson's *r*), indicating how they were calculated

Our web collection on statistics for biologists contains articles on many of the points above.

Software and code

Policy information about availability of computer code

Data collection	Data provided from analysis platform as MS Excel files.
Data analysis	R version 3.4.2 (2017-09-28) with additional packages as described in the paper. Notably 'caret', 'pROC', 'glmnet' and 'beanplot'

For manuscripts utilizing custom algorithms or software that are central to the research but not yet described in published literature, software must be made available to editors/reviewers. We strongly encourage code deposition in a community repository (e.g. GitHub). See the Nature Research guidelines for submitting code & software for further information.

Data

Policy information about availability of data

All manuscripts must include a data availability statement. This statement should provide the following information, where applicable:

- Accession codes, unique identifiers, or web links for publicly available datasets
- A list of figures that have associated raw data
- A description of any restrictions on data availability

Complete measurements (including individual replicates) for the final cohort will be made available here https://doi.org/10.6084/m9.figshare.7642268 upon publication.

Field-specific reporting

Please select the one below that is the best fit for your research. If you are not sure, read the appropriate sections before making your selection.

☒ Life sciences ☐ Behavioural & social sciences ☐ Ecological, evolutionary & environmental sciences

Life sciences study design

All studies must disclose on these points even when the disclosure is negative.

Sample size	All available samples in the biobanks used with the sought-after diagnoses were collected. No power-calculations were carried out before hand. This is discussed in the manuscript. Based on prior publications and similar set-ups, the samples sizes aquired were deemed sufficient.
Data exclusions	If abundance levels for a specific protein was found to be below level of detection (LOD) for all samples, this protein was excluded. Otherwise measurements below limit of detection was replaced with the LOD.
Replication	Models were generated in a discovery cohort and performance was then estimated in 2 or 3 replication cohorts. Similiar performances was obtained in the replication cohorts.
Randomization	Samples were kept in groups based on cohort and diagnosis, but randomized across plates for protein characterization allowing for normalization of any cohort-specific biases.
Blinding	No blinding was used. We apply supervised machine-learning to generate the models and therefore need to know the outcome.

Reporting for specific materials, systems and methods

We require information from authors about some types of materials, experimental systems and methods used in many studies. Here, indicate whether each material, system or method listed is relevant to your study. If you are not sure if a list item applies to your research, read the appropriate section before selecting a response.

Materials & experimental systems

n/a	Involved in the study
☒	☐ Antibodies
☒	☐ Eukaryotic cell lines
☒	☐ Palaeontology
☒	☐ Animals and other organisms
☐	☒ Human research participants
☒	☐ Clinical data

Methods

n/a	Involved in the study
☒	☐ ChIP-seq
☒	☐ Flow cytometry
☒	☐ MRI-based neuroimaging

Human research participants

Policy information about studies involving human research participants

Population characteristics	All female samples diagnosed with suspicion of ovarian cancer diagnosed surgically with either benign, borderline or malign (stage I-IV) conditions. Age at diagnosis and clinically measured CA-125 was also available. For some of the women additional clinical information was available but not used in the study. No genotypic information was available.
Recruitment	Plasma samples was collected from Swedish biobanks. No specific recruitment for this study was carried out.
Ethics oversight	The study was approved by the Regional Ethics Committees in Uppsala (Dnr: 2016/145) and Göteborg (Dnr: 201-15).

Note that full information on the approval of the study protocol must also be provided in the manuscript.
